# Development of a Population Pharmacokinetic Gabapentin Model Leveraging Therapeutic Drug Monitoring Concentrations

**DOI:** 10.3390/pharmaceutics16121514

**Published:** 2024-11-25

**Authors:** Firas Al-Zubaydi, Andrew Wassef, Leonid Kagan, Luigi Brunetti

**Affiliations:** 1 Department of Pharmacy Practice, Ernest Mario School of Pharmacy, Rutgers, The State University of New Jersey, 160 Frelinghuysen Road, Piscataway, NJ 08854, USA; firas.jawad@copharm.uobaghdad.edu.iq; 2 Department of Pharmaceutics, College of Pharmacy, University of Baghdad, Baghdad 10071, Iraq; 3 Department of Pharmaceutics, Ernest Mario School of Pharmacy, Rutgers, The State University of New Jersey, 160 Frelinghuysen Road, Piscataway, NJ 08854, USA; awassef@scarletmail.rutgers.edu (A.W.); lkagan@pharmacy.rutgers.edu (L.K.); 4 Center of Excellence in Pharmaceutical Translational Research and Education, Ernest Mario School of Pharmacy, Rutgers, The State University of New Jersey, Piscataway, NJ 08854, USA; 5 Robert Wood Johnson University Hospital Somerset, 110 Rehill Avenue, Somerville, NJ 08876, USA

**Keywords:** gabapentin, gamma-aminobutyric acid, population pharmacokinetics, therapeutic drug monitoring, creatinine

## Abstract

**Background/Objectives:** Gabapentin has variable pharmacokinetics (PK), which contributes to difficulty in dosing and increased risk of adverse events. The objective of this study was to leverage gabapentin concentrations from therapeutic drug monitoring (TDM) to develop a population PK (popPK) model and characterize significant covariates that impact gabapentin PK. **Methods:** Data were retrospectively collected from 82 hospitalized adult patients with TDM gabapentin concentrations. Renal function indicators (i.e., estimated glomerular filtration rate, creatinine clearance, acute kidney injury), body weight parameters (i.e., actual body weight, ideal body weight, adjusted body weight, lean body weight, body mass index, obesity status), fasting plasma glucose levels, and diagnosis of type 2 diabetes were tested as potential covariates. A popPK model was developed in MONOLIX (2020R1, Lixoft, France). **Results**: A one-compartment model best described gabapentin PK with first-order absorption, dose-dependent bioavailability, first-order elimination, and no lag time. Population parameter estimates for the volume of distribution (V_d_), and clearance (Cl) were 44.61 L, and 5.73 L/h, respectively. Serum creatinine was a significant covariate on Cl. **Conclusions**: The popPK model highlights the importance of renal function in the interindividual variability of gabapentin PK and suggests that diabetes and body weight parameters have no impact on gabapentin PK. Moreover, our study supports the utility of leveraging data obtained from clinical TDM for popPK model development.

## 1. Introduction

Gabapentin [1-(aminomethyl)-cyclohexane acetic acid] is an antiepileptic drug with analgesic properties. The drug was first approved by the Food and Drug Administration (FDA) in 1993 [1]. Gabapentin is indicated to treat partial-onset seizures and postherpetic neuralgia and is often used off-label for the treatment of diabetic neuropathy [1,2,3,4]. As a structural analog of gamma-aminobutyric acid (GABA), gabapentin was designed to be more lipophilic than GABA and cross the blood–brain barrier more readily [1,5]. The precise mechanism of gabapentin’s antileptic and analgesic effects is currently unknown [1]. Gabapentin is actively absorbed in the gut via the L-amino acid transporter system [5,6,7]. Food has minimal effects on the rate and extent of its absorption [1,5,6]. Gabapentin is largely unbound to plasma proteins [1]. Peak plasma concentration (C_max_) is achieved within 2–3 h after oral administration. Plasma concentrations are dose-proportional across typical clinical ranges (300 mg to 400 mg every 8 h), ranging between 1 µg/mL and 10 µg/mL, but are less than dose-proportional at higher dose levels (>600 mg every 8 h) [5,6,7]. Gabapentin does not undergo metabolism by liver enzymes and is excreted renally as an unchanged drug with a half-life of 5–7 h [1,5,6,7].

The high interindividual variability of gabapentin pharmacokinetics (PK) contributes to difficulty in reaching effective doses and an increased risk of adverse events [6,7,8,9,10]. The total daily dose of gabapentin is correlated with incidence rates of side effects such as dizziness and somnolence, which negatively impacts patient adherence to therapy [10]. Therefore, identifying covariates that impact gabapentin PK can help guide dose selection and improve the safety and tolerance of gabapentin.

Diabetes mellitus may lead to alterations in drug PK, but there is limited clinical guidance for dosage adjustment in this patient population [11,12]. One of the complications of diabetes is the delayed gastric emptying time; therefore, the absorption rate of orally administered medication may be changed [12]. About 32–47% of untreated patients with type 2 diabetes experience delayed gastric emptying [13].

Theoretically, drugs with carrier-mediated transporter systems are more sensitive to longer gut transit time than drugs absorbed via passive diffusion [11]. Another long-term sequelae of diabetes is altered renal functions, with normal or increased glomerular filtration rate (GFR) during the first five to ten years of the disease and significantly reduced GFR as diabetic nephropathy progresses [11,12,14]. Nephropathy is present in up to 20–40% of patients with diabetes [14]. The volume of distribution can also be altered in patients with diabetes. Chronic hyperglycemia can lead to protein glycation and changing concentration in the circulating free fatty acids, leading to consequent changes in the fraction of unbound drugs [11,12]. The volume of distribution also correlates with the degree of obesity, a common comorbidity in patients with type 2 diabetes [15].

Previous publications have consistently highlighted biomarkers for renal function as a significant covariate on the oral clearance of gabapentin. Creatinine clearance (CrCl) was reported to be linearly correlated with the apparent oral clearance of gabapentin [5,16,17]. Gabapentin elimination is affected directly by age- or disease-related decreases in renal function [6]. However, there is no consensus regarding the effect of diabetes and obesity on gabapentin PK. Ouellet et al. suggested that body weight is a significant covariate on the volume of distribution of gabapentin in the pediatric population. In contrast, other PK studies performed in the adult population did not include body weight in the final model [16,18,19,20]. A non-compartmental analysis (NCA) by Costa et al. reported that patients with type 2 diabetes exhibited decreased gabapentin C_max_, increased clearance, and increased volume of distribution compared with the control group [21]. Costa et al. later developed a popPK model, which showed no significant effect of diabetes or hyperglycemia on the PK profile of gabapentin [19]. The paucity in the literature and the conflicting results indicate a need to better characterize covariates for gabapentin PK.

Moreover, previous models were developed using data collected in prospective controlled studies with frequent sampling, which can be resource-intensive. On the other hand, clinical therapeutic drug monitoring (TDM) levels are readily available and may allow for the development of popPK models without additional blood sampling [22,23]. Clinicians have frequently employed TDM to optimize treatment for drugs with a poor correlation between dose and clinical efficacy and in special patient populations with altered PK [24,25]. Patients with diabetes and obese individuals have been proposed to be beneficiaries of TDM [26,27,28]. This study aimed to develop a popPK model of gabapentin disposition using real-life clinical TDM data and to evaluate the effect of covariates, including diabetes and obesity status, on pharmacokinetic parameters.

## 2. Methods

### 2.1. Study Population and Data Collection

The study protocol was approved by the investigational review boards of Rutgers University and Robert Wood Johnson University Hospital Somerset (RWJS), New Jersey, USA. Data were retrospectively collected from patients with available TDM gabapentin concentrations. Serum gabapentin concentrations were extracted from the electronic health records of all consecutive patients from 1 January 2009 to 7 December 2023. The study included all adult patients (≥18 years) who had received at least one oral dose of gabapentin after admission and had at least one plasma concentration collected after the dose. Pregnant patients and children (<18 years) were excluded. Patients were either taking gabapentin at home or initiated on gabapentin during the hospital stay. The medical indication for gabapentin was not available in the dataset but represents a random general population. The date and time of gabapentin administration before the sample collection and the time of sample collection were recorded. Patients’ demographic and clinical characteristics were collected for covariate analysis (Table 1). Lean body weight was calculated using the Janmahasatian equation [29]. Estimated glomerular filtration rate (eGFR) was calculated using the Modification of Diet in Renal Disease (MDRD) equation [30]. Obesity was defined by a body mass index (BMI) of greater than or equal to 30 kg/m^2^. The diagnosis of type 2 diabetes was determined based on the admission notes.

### 2.2. Bioanalytical Methods

Plasma gabapentin concentrations were quantitated under a service agreement between the medical center and the Mayo Clinical Laboratory using a liquid chromatography–tandem mass spectrometry method [31]. The laboratory determined the assay’s performance characteristics to be consistent with Clinical Laboratory Improvement Amendments requirements. The reference range was determined to be 2.0–20.0 µg/mL with a concentration of ≥25 µg/mL being considered the toxic concentration.

### 2.3. Population Pharmacokinetic Analysis

A popPK model was developed in MONOLIX (2020R1, Lixoft, Antony, France) using the stochastic approximation expectation maximization (SAEM) algorithm. The model-building process involves identifying a structural model, optimizing the error model, and assessing covariates.

A one-compartment model with linear elimination was used to describe the disposition of gabapentin, and different absorption models were evaluated, including first-order absorption with or without lag time (T_lag_) and transit compartment absorption models (Table 2). Both linear and nonlinear bioavailability models were tested for first-order absorption models. The following equation was applied to describe dose-dependent bioavailability, and the parameters were fixed to previously published values [20]:

(1) $$F=\frac{D_{max}}{D_{50}+Dose}$$

where F is gabapentin bioavailability, $D_{max}\;(823\;mg/day)$ is the maximal absorption rate, $D_{50}\;(1120\;mg/day)$ is the dose when the absorption process is 50% saturated, and Dose is the last gabapentin dose before the sample collection [20]. Initial values for the population parameters were set to previously reported values. The limited number of concentrations obtained during the absorption phase can undoubtedly cause high variability with the population estimate for the absorption rate constant (k_a_) and the delay time (T_lag_). Therefore, values of 0.778 h^−1^ and 0.31 h were considered as fixed values for k_a and_ T_lag_, respectively [16,32]. Models were evaluated based on the likelihood results (i.e., objective function value (OFV), Akaike information criterion (AIC), and Bayesian information criterion (BIC)), relative standard errors (RSEs) of parameters, visual examinations (i.e., visual predictive check (VPC), observation versus prediction plot), and model stability. Structural model stability was assessed in five convergence assessment runs using the SAEM algorithm. Estimated population parameters with high variability were fixed to improve model stability. A constant error model was selected based on the proposal created by MONOLIX.

Both continuous and categorical covariates were assessed in the covariate model. Continuous covariates include age, body weight parameters (i.e., actual body weight (WT), ideal body weight (IBW), adjusted body weight (ABW), lean body weight (LBW), BMI), renal function indicators (i.e., eGFR, CrCl, SCr), and fasting plasma glucose levels (FPG). The serum creatinine value from the day of gabapentin concentration collection was used to calculate renal function indicators. Categorical covariates include sex, diagnosis of type 2 diabetes, presence of acute kidney injury (AKI), obesity status, and a combined covariate of obesity and diabetes (i.e., patients were categorized into diabetic but not obese, obese but metabolically healthy, both diabetic and obese, and non-diabetic and non-obese). All continuous covariates were log-transformed and centered using mean values. Covariates were selected based on three clinical questions in the following order: the effects of renal function on the clearance (Cl), the effects of body size metric on the apparent volume of distribution (V_d_), and the effects of diabetes on absorption rate constant (k_a_) and V_d_. After all the covariates relevant to the clinical questions were tested, any additional covariates that were significantly correlated (*p* ≤ 0.01) with random effects were tested. The covariate was added if the addition of a covariate yielded a reduction of 3.84 or greater in the OFV (*p* ≤ 0.05) and if the resulting β coefficient was significantly (*p* ≤ 0.01) different from 0 (based on the Wald test and correlation test). Once all covariates were added, a backward deletion was performed. The model retained a covariate if the deletion increased by 6.63 in the OFV (*p* ≤ 0.01). VPC, goodness-of-fit plots, RSE, and likelihood results were also used to assess the best model. A bootstrap analysis with 1000 replicates was applied to validate the stability and robustness of the final popPK model.

## 3. Results

### 3.1. Participants

A total of 123 gabapentin TDM concentrations from 108 patients were available. Gabapentin TDM concentrations ranged from 0.6 to 56.2 µg/mL (7.97 ± 7.8, mean ± SD). Out of 108 patients, 82 fit the inclusion and exclusion criteria, and their plasma concentrations were used to build the popPK model. The study population was predominantly white, with an average age of 65.7 years and an average BMI of 30 (Table 1). Eighteen (22%) patients had a BMI of greater than 30 and were thus considered obese. Twenty-six (31.7%) patients had a diagnosis of type 2 diabetes. The cohort with diabetes had a higher BMI, higher body weight, higher FPG, lower eGFR, and were older. A total of 18 (21.9%) patients experienced AKI within 48 h of the sample collection. Single gabapentin dosages ranged from 100 mg to 1200 mg (median, 300 mg).

### 3.2. Population Pharmacokinetic Modeling

A one-compartment model with first-order absorption, no lag time, nonlinear bioavailability, and linear elimination provided a good description of the data and, therefore, was selected as the base structural model. Other potential absorption models were tested but did not provide a significant improvement (Table S1). Moreover, most of the study population received a dose equal to or less than 400 mg, during which dose-proportionality was established [6]. Due to the lower number of concentrations in the dataset, especially in the drug’s absorption phase, a simple first-order absorption model with a fixed absorption rate constant (k_a_) value (0.778 h^−1^) was selected over other more complex absorption models. The dose-dependent bioavailability model decreased by 10.98 in OFV when compared with the dose-independent bioavailability model (496.44 versus 507.42), and it was selected as the superior model (Table S1).

Because dose adjustment of gabapentin depends on the patient’s renal function, the first step was to assess the effect of renal function on gabapentin clearance. CrCl, SCr, eGFR, and the presence of AKI were tested separately as a covariate on Cl. SCr was selected as a significant covariate on Cl (Table S2). Next, the effect of obesity or body weight on V_d_ was assessed: WT, IBW, ABW, LBW, BMI, and obesity status were tested separately. None of the covariates resulted in a statistically significant drop in OFV and, therefore, were not included in the model. After that, a diagnosis of diabetes, FPG, and the combined covariate of obesity and diabetes status were tested separately as covariates on k_a_ and V_d_. Similarly, none of the covariates resulted in a statistically significant drop in OFV and, therefore, were not included in the model. After adding SCr to the model, no additional covariates were significantly (*p* ≤ 0.01) correlated with random effects.

**Figure 1:**
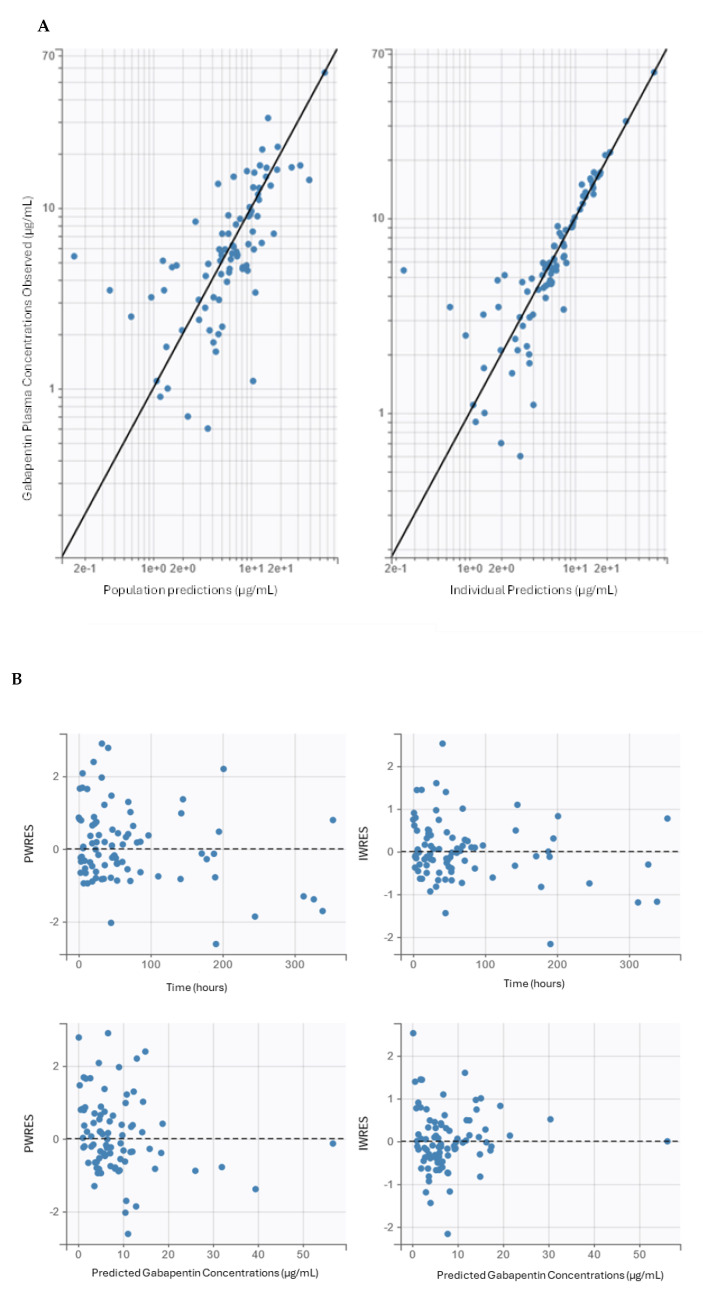

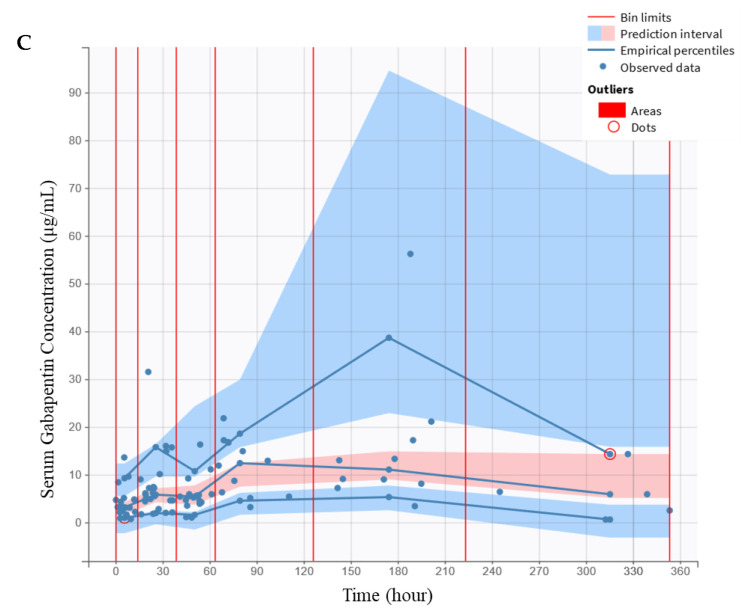
(**A**) Observation versus prediction plots for population and individual predictions. The black linear line is the y = x line. (**B**) Population and individual weighted residuals (PWRESs and IWRESs) plotted against time from the first recorded gabapentin dose (upper panels) and predicted gabapentin concentrations (bottom panels). (**C**) Visual predictive check (VPC) plot for the final model. Observed data are represented by blue dots. The black lines represent the 5th, 50th, and 95th percentiles of the predicted value. The blue areas represent the 95% confidence interval of the predicted percentile. Time 0 was the time of the first recorded gabapentin dose for each individual subject.

Population parameter estimates for V_d_ and Cl were 44.61 L and 5.73 L/h, respectively (Table 2). SCr was included as a significant covariate on Cl. Goodness-of-fit plots showed alignment between observed and predicted gabapentin concentrations (Figure 1A). Individual conditional weighted residuals (IWRESs) were normally distributed and centered around zero (Figure 1B). Plots of IWRESs against time and concentration were evenly centered around zero. Despite the decrease in the model’s predictability at low gabapentin concentrations and early time points, the final popPK model demonstrated acceptable adequacy (Figure 1B). As shown in Table 2, the bootstrap analysis resulting from 1000 replicates of the final popPK model validates acceptable robustness and precision. Shrinkage was <5% for all parameters. VPC also demonstrated that simulated data are consistent with observed data (Figure 1C).

## 4. Discussion

This study aimed to explore the utility of TDM concentrations in developing a nonlinear mixed-effect PK model and characterize covariates that affect the PK profile of gabapentin, with a particular interest in diabetes and obesity. Our model suggested that gabapentin PK was best described by a simple one-compartment model with first-order absorption with nonlinear bioavailability and first-order elimination, with SCr as a covariate on Cl.

The population estimate for V_d_ is approximately similar to the previously reported value (45.4 L) [32]. The population estimate for Cl by this model is smaller than the previously reported values (6.31 L/h) [32]. The inclusion of SCr as a covariate on clearance confirmed the importance of renal function in gabapentin elimination. Previous studies included either CrCl or eGFR as a covariate on clearance in their final popPK models for gabapentin [16,18,19,20]. Gabapentin is predominately renally unchanged when excreted (77%) with first-order elimination PK, and a dose reduction of greater than 50% is recommended for adult patients with a CrCl of less than 60 mL/min [1,5]. In addition, oral clearance of gabapentin was reported to be linearly correlated with CrCl [5,16,17,18]. During the model development, when SCr was compared with CrCl and eGFR as a covariate on clearance, SCr resulted in a significant reduction in OFV. Plasma clearance and renal clearance of gabapentin are directly proportional to the patient’s creatinine clearance due to its primarily renal elimination [5,16,17].

In our model, the cohort with diabetes or hyperglycemia had no impact on the PK of gabapentin, and this finding was consistent with previously published popPK models for gabapentin in the adult population with type 2 diabetes [19]. Because patients with type 2 diabetes tend to have a heavier body weight, our model also investigated different body weight parameters as covariates. WT, LBW, ABW, and IBW were evaluated but not included in the final model. This finding was consistent with previously published popPK models for gabapentin in the adult population [16,19,20]. Ouellet et al. concluded that the volume of distribution was related to body weight in their popPK model for gabapentin. However, the study was performed in the pediatric population, with a distinct PK profile from the adult population [18]. Overall, body weight seems to have a minimal effect on gabapentin PK and therefore is not a significant factor for dosing consideration.

Due to gabapentin’s considerable interindividual variations in its PK and pharmacodynamics, it has been suggested that TDM may be advantageous. The absence of gabapentin therapeutic effect in certain patients might be due to inadequate drug plasma concentration [8]. In contrast, the high plasma concentrations of gabapentin could result in common adverse effects, such as dizziness and sleepiness, which can significantly impact the well-being and compliance of patients [4,5]. Therefore, TDM could be beneficial in achieving the desired effective drug concentrations and lowering the possibility of adverse effects. The PopPK model developed in this study can describe the interpatient variability of gabapentin by correlating Cl with SCr and the dose-dependent bioavailability. This model can be applied to the prediction of the drug concentration for better individual dose optimization.

Our findings characterized our study population well while confirming several conclusions drawn by previous studies. However, this study has some limitations. Firstly, due to the study’s retrospective nature, consistencies and human errors can happen with the sample collection and record documentation. Secondly, our sample size is relatively small as gabapentin TDM concentrations are not routinely collected. We acknowledge residual confounding factors may be present; however, as an observational study, data were limited to what was accessible in the medical record. Further investigation with a larger sample size may allow for a more comprehensive examination of the influence of additional covariates.

The use of TDM concentrations to develop popPK models has been implemented across various disease states and drug classes. This approach has several benefits, including utilizing existing data, conserving resources, and reducing financial burdens. Additionally, employing a real-world population can enhance the external validity of the model. Available concentration data can be used to create a new model or to refine existing published models. In this study, we elected to create a new model due to the differences in the populations used in previously published gabapentin popPK models. Our goal was to focus on developing a model that is applicable to a broad population. While utilizing available gabapentin concentrations from clinical care was a resource-efficient approach, some limitations must be acknowledged in this approach. The development process relies on the available data, which may not cover the entire dosing interval. This can hinder our ability to accurately capture the absorption rate constant (Ka) or the elimination rate constant (Ke). However, we can fix these variables if there are existing data that describes them, as we have performed. Concerns regarding the use of existing data include limited dose ranges, restricted indications, underrepresentation of different races and ethnicities, and general assumptions, such as the achievement of a steady-state concentration. Taylor and colleagues have provided a concise review of important considerations for developing a popPK model [33]. Despite these limitations, a valuable model can still be created if these issues are addressed during its development and application.

In summary, this study characterized gabapentin PK and demonstrated the potential of repurposing TDM concentrations to develop popPK models, thus addressing the paucity of large-scale prospective PK studies in patients. Previously published gabapentin popPK models in adult populations were based on data collected in prospective PK studies. Our model based on TDM concentrations confirmed the major findings from these prospective studies [16,19,20]. This study demonstrated that TDM concentrations could be utilized to develop popPK models and investigate the unique PK alterations of gabapentin in patients.

## 5. Conclusions

Gabapentin TDM concentrations were used to develop a popPK model for gabapentin. Gabapentin PK was described by a one-compartment model with first-order absorption with nonlinear bioavailability and first-order elimination. The interindividual variability of gabapentin PK was associated with SCr in our study population. Obesity and diabetes were not significant covariates in the popPK model. The results of this study reinforce the importance of considering renal function when dosing gabapentin.

## Figures and Tables

**Table 1:** Demographic and clinical characteristics of individuals included in the analysis (n = 82).

		Mean ± SD	Range
Age (year)		65.7 ± 16.4	22–93
Actual body weight (kg)		84.3 ± 25.9	44.2–195.0
Ideal body weight (kg)		59.9 ± 10.3	43.2–82.2
Adjusted body weight (kg)		69.7 ± 14.3	45.0–123.0
Lean body weight (kg) ^a^		54.9 ± 13.4	30.5–91.9
Height (cm)		167.1 ± 9.8	149.9–187.9
BMI (kg/m^2^)		30.0 ± 7.9	18–60
Serum creatinine (0.66–1.25 mg/dL)		1.3 ± 1.0	0.4–3.8
eGFR (mL/min/1.73m^2^) ^b^		71.0 ± 42.4	16.0–127
Creatinine clearance (mL/min) ^c^		91.67 ± 67.6	13.3–192.7
Fasting plasma glucose (mg/dL)		127.6 ± 78.2	76.0–251.0
Serum concentration (μg/mL)		7.97 ± 7.8	0.6–56.2
ALT (0–49 U/L)		30.8 ± 41.6	8.0–245.0
AST (17–59 U/L)		30.9 ± 44.4	6.0–74.0
Total bilirubin (0.2–1.3 mg/dL)		0.57 ± 0.7	0–2.7
Albumin (3.5–5 g/dL)		3.7 ± 0.7	1.4–4.6
		Median	Range
Single dose amount (mg) ^d^		300	100–1200
Total daily dose (mg) ^e^		900	100–2700
		n (%)	
Sex	Male	31 (37.8)	
	Female	51 (62.2)	
Race	White	73 (89.0)	
	Asian	1 (1.2)	
	Black	6 (7.3)	
	Other	2 (2.4)	
Type 2 diabetes	Yes	26 (31.7)	
Presence of AKI ^f^	Yes	18 (21.9)	
Obesity ^g^	Yes	18 (22.0)	
Combined covariate of obesity and diabetes	Obesity + diabetes	8 (9.8)	
	Obesity only	22 (26.8)	
	“Healthy”	52 (63.4)	

ALT: Alanine aminotransferase; AKI: acute kidney injury; AST: aspartate aminotransferase; BMI: body mass index; eGFR: estimated glomerular filtration rate; SD: standard deviation. ^a^ Lean body weight was calculated using the Janmahasatian formula. ^b^ eGFR was calculated using the Modification of Diet in Renal Disease (MDRD) equation. ^c^ Creatinine clearance was calculated with the Cockcroft–Gault formula. The weight factor was adjusted to ideal body weight when the actual body weight was greater or equal to 30% of the ideal body weight. ^d^ Single-dose amount was defined by the last given dose. ^e^ Total daily dose was defined by the accumulated total dose over the past 24 h prior to the sample collection. ^f^ Acute kidney injury was defined by a change in serum creatinine of more than 0.3 mg/dL in the past 48 h. ^g^ Obesity was defined by BMI ≥ 30.

**Table 2:** Final model population parameter estimates and bootstrap analysis.

Parameters	Population Estimates (RSEs (%))	Bootstrap Analysis Median (95%CI)
Fixed effects		
k_a_ (h^−1^)	0.778 (fixed)	0.778 (fixed)
V_d_ (L)	44.61 (15.94)	45.99 (32.84–65.91)
Cl (L/h)	5.73 (17.62)	7.01 (4.3–12.54)
β _Cl_SCR_	−0.89 (16.94)	−1.12 ((−1.66)–(−0.58))
Random effects		
ω _Vd_	0.77 (19.98)	0.75 (0.42–1.07)
ω _Cl_	0.28 (28.59)	0.26 (0.11–0.48)
Error model parameter		
a	2.03 (17.5)	1.93 (1.1–2.65)

Cl: Clearance; k_a_: absorption rate constant; RSE: relative standard error; V_d_: volume of distribution; a: constant error model; ω: standard deviation.

## Data Availability

The data that support the findings of this study are available from the corresponding author upon reasonable request.

## Supplementary Materials

The following supporting information can be downloaded at: https://www.mdpi.com/article/10.3390/pharmaceutics16121514/s1, Table S1: Summary of structural model selection; Table S2: Summary of the covariate model building process.
