# Supplementary material for: Development of a Population Pharmacokinetic Gabapentin Model Leveraging Therapeutic Drug Monitoring Concentrations
Source: Pharmaceutics. 2024 Nov 25;16(12):1514. doi: 10.3390/pharmaceutics16121514 (PMC11678411; doi:10.3390/pharmaceutics16121514)
Supplement: Supplementary file 1 [file pharmaceutics-16-01514-s001.zip › pharmaceutics-3271482-supplementary.pdf]

Table S1. Summary of structural model selection.

Models	1st-order absorption with no T_{lag}	1st-order absorption with no T_{lag} and nonlinear F	1st-order absorption with T_{lag}	1st-order absorption with T_{lag} and nonlinear F	Transit compartment
**Likelihood results**					
-2*LL (IS)	507.42	496.44	508.91	499.57	505.39
BICc (IS)	529.56	517.81	535.46	526.11	545.23
Fixed effects (RSE)					
k_a	0.778 (fixed%)	0.778 (fixed)	0.778(fixed)	0.778 (fixed)	0.778 (fixed)
V_d	80.95 (27.3%)	43.76 (18.0%)	64.14 (22.8%)	32.11 (20.3%)	35.86 (161%)
Cl	2.74 (18.3%)	1.59 (16.4%)	3.61 (15.7%)	1.93 (13.4%)	2.41 (26.4%)
T_{lag}			0.31 (fixed)	0.31 (fixed)	
K_{tr}					0.13 (41.9%)
Mtt					8.42 (14.7%)
D_{max}		823 (fixed)		823 (fixed)	
D_{50}		1120 (fixed)		1120 (fixed)	
Standard deviation (RSE)					
ω_{Vd}	0.76 (15.3%)	0.75 (18.8%)	0.8 (19.1%)	0.72 (21.0%)	0.86 (79.2%)
ω_{Cl}	1.06 (16.2%)	0.84 (13.5%)	0.86 (13.6%)	0.81 (13.8%)	0.82 (22.3%)
ω_{Ktr}					0.36 (74.0%)
ω_{Mtt}					0.24 (51.9%)
Residual error parameters (RSE)					
a	1.24 (71.9%)	1.64 (21.6%)	2.09 (20.9%)	1.79 (26.7%)	2.36 (NaN)

BIC: Bayesian information criterion; Cl: clearance; F: bioavailability; IS: importance sampling; k_a: absorption rate constant; K_{tr}: transit rate between compartments; Mtt: mean transit time; D_{max} : the maximal absorption rate; D50 : the dose when the absorption process is 50% saturated; RSE: relative standard error; T_{lag}: lag time; V_d: volume of distribution; V_m: maximum absorption rate; -2*LL: log-likelihood; a : constant error model; ω : standard deviation.

Table S2. Summary of the covariate model building process.

No.	Description	-2*LL (IS)	Δ-2LL	Wald Test
	1st-order absorption with no T_{lag} and nonlinear F (one compartment with constant error model and fixed k_a)	496.44		
	Effects of renal function	*Compared with base model*		
1	Base model + SCr on Cl	452.85	-43.59••	p<0.01
2	Base model + CrCl on Cl	475.74	-20.70••	p<0.01
3	Base model + eGFR on Cl	461.20	-35.24••	p<0.01
4	Base model + AKI on Cl	482.61	-13.83••	p<0.01
	Effects of body weight	*Compared with model 1*		
5	1 + IBW on V_d	457.20	4.35	p<0.05
6	1 + WT on V_d	456.83	3.98	p<0.05
7	1 + ABW on V_d	456.41	3.56	NS
8	1 + LBW on V_d	457.44	4.59	p<0.05
9	1 + BMI on V_d	457.24	4.39	p<0.05
	Effects of diabetes	*Compared with model 1*		
10	1 + DM on Vd	456.83	3.98	p<0.05
11	1 + FPG on Vd	456.77	3.92	p<0.05
12	1 + Obesity _ DM on V_d	456.49	3.64	NS
	**Backward deletion**	**Compared with model 1**		
13	Final model – SCr on CL	495.90	43.05**	

ABW: Adjusted body weight; BMI: body mass index; Cl: clearance; CrCl: creatinine clearance; DM: diagnosis of type 2 diabetes; eGFR: estimated glomerular filtration rate; FPG: fasting plasma glucose levels; IBW: ideal body weight; IS: importance sampling; LBW: lean body weight; NS: not statistically significant (p>0.1); Obesity _DM: combined covariate of diabetes and obesity (i.e., patients were categorized into diabetic but not obese, obese but metabolically healthy, both diabetic and obese, and non-diabetic and non-obese); V_d: volume of distribution; -2*LL: log likelihood; Δ-2LL: change in log likelihood; WT: actual body weight. *p<0.05, **p<0.01.
